# Risk of suicide after suicide attempt according to coexisting psychiatric disorder: Swedish cohort study with long term follow-up

**DOI:** 10.1136/bmj.a2205

**Published:** 2008-11-18

**Authors:** Dag Tidemalm, Niklas Långström, Paul Lichtenstein, Bo Runeson

**Affiliations:** 1 Department of Clinical Neuroscience, Karolinska Institutet, Division of Psychiatry, St Göran, SE-112 81 Stockholm, Sweden; 2 Centre for Violence Prevention, Karolinska Institutet; 3 Department of Medical Epidemiology and Biostatistics, Karolinska Institutet; 4 Department of Clinical Neuroscience, Karolinska Institutet

## Abstract

**Objective** To investigate the impact of coexistent psychiatric morbidity on risk of suicide after a suicide attempt.

**Design** Cohort study with follow-up for 21-31 years.

**Setting** Swedish national register based study.

**Participants** 39 685 people (53% women) admitted to hospital for attempted suicide during 1973-82.

**Main outcome measure** Completed suicide during 1973-2003.

**Results** A high proportion of suicides in all diagnostic categories took place within the first year of follow-up (14-64% in men, 14-54% in women); the highest short term risk was associated with bipolar and unipolar disorder (64% in men, 42% in women) and schizophrenia (56% in men, 54% in women). The strongest psychiatric predictors of completed suicide throughout the entire follow-up were schizophrenia (adjusted hazard ratio 4.1, 95% confidence interval 3.5 to 4.8 in men, 3.5, 2.8 to 4.4 in women) and bipolar and unipolar disorder (3.5, 3.0 to 4.2 in men, 2.5, 2.1 to 3.0 in women). Increased risks were also found for other depressive disorder, anxiety disorder, alcohol misuse (women), drug misuse, and personality disorder. The highest population attributable fractions for suicide among people who had previously attempted suicide were found for other depression in women (population attributable fraction 9.3), followed by schizophrenia in men (4.6), and bipolar and unipolar disorder in women and men (4.1 and 4.0, respectively).

**Conclusion** Type of psychiatric disorder coexistent with a suicide attempt substantially influences overall risk and temporality for completed suicide. To reduce this risk, high risk patients need aftercare, especially during the first two years after attempted suicide among patients with schizophrenia or bipolar and unipolar disorder.

## Introduction

A suicide attempt is a risk factor for completed suicide; the absolute risk in people followed-up for 5-37 years was 7-13%,^1^ ^2^ ^3^ ^4^ ^5^ ^6^ roughly corresponding to a 30-40 times increased risk of death from suicide in those who had attempted suicide compared with the general population.^7^ These figures, however, suggest that suicide is a comparatively rare event even in a high risk group of people who have attempted suicide. It is therefore vital to identify those at highest risk of completed suicide in this group.

The impact of coexistent psychiatric morbidity on risk of suicide after suicide attempts is largely unknown. Limited research suggests that coexisting substance misuse, especially in young men,^1^ and major depression^8^ increases the risk of suicide in those who have attempted suicide. One study of a large Finnish cohort^9^ found that pharmacological treatment of a depressive disorder decreased the risk of suicide after a suicide attempt. Another trial^10^ studied the prevalence of different psychiatric disorders in people who had attempted suicide but not with completed suicide as outcome measure. Researchers studied death from suicide in different diagnostic groups in the general population of Denmark but did not take suicide attempts into account.^11^ Consequently the relation of coexistent psychiatric morbidity with risk of completed suicide in people who have attempted suicide needs to be examined more closely.^12^ ^13^

The interval between first communication of suicidal ideation or suicidal behaviour and completed suicide varies with coexisting mental disorder; it is reportedly shorter in depressive disorders than in personality and psychotic disorders.^14^ Furthermore, the time from onset of affective disorder to completed suicide seems to vary according to subtype of affective disorder.^15^ These findings suggest that the interval from suicide attempt to completed suicide should be studied further.

To inform clinical psychiatric and suicide prevention practices, we studied a cohort of 39 685 people admitted to hospital as a result of suicide attempts in Sweden during 1973-82. We linked data from the hospital discharge register and the cause of death register and followed-up the people longitudinally for up to 31 years until 2003. Firstly, we explored possible temporal differences in the occurrence of completed suicide across different diagnostic categories. Then we investigated if the risk for completed suicide varied with psychiatric disorder in those who had attempted suicide. Finally, we calculated the population attributable fraction,^16^ an estimate of the percentage of suicides among people who had previously attempted suicide, that would not have happened without each studied coexistent psychiatric disorder.

## Methods

Using the unique national identification number assigned to all Swedish citizens we linked data on all people living in Sweden during 1973-82 (n=9.4 million) to the hospital discharge register (held by the Epidemiological Centre of the National Board of Health and Welfare), cause of death register (Epidemiological Centre), 1970 population and housing census, and education and migration registers (the last three held by Statistics Sweden).

We then identified people aged 10 or older who had been admitted to inpatient care in Sweden during 1973-82 because of suicidal behaviour, defined as a definite suicide attempt (E950-9, international classification of diseases, eighth revision) or an uncertain suicide attempt (E980-9), as registered in the hospital discharge register (n=49 509). In case of more than one admission for a suicide attempt we used the first admission as the index attempt. To avoid confounding effects of being in the asylum seeking process we excluded people who had immigrated within two years before baseline (n=860). We identified cases as those with one of the studied psychiatric diagnoses present at discharge from the index admission for a suicide attempt or at discharge from the first inpatient episode beginning within one week after this index episode, as recorded in the hospital discharge register (n=12 681). Those without a diagnosis of mental disorder within one year after the suicide attempt were used as reference subjects (n=27 004). We did not include people with a different psychiatric diagnosis to those studied, or a diagnosis after one week but within one year after the suicide attempt (n=8964). The study cohort thus consisted of 39 685 people; 18 642 males and 21 043 females, mean age 38.4 (SD=16.5) years and 37.0 (SD 17.0) years, respectively.

The reference group comprised 68% of the study cohort. Reference subjects had attempted suicide but had no coexistent mental disorder within one year after the attempt. In clinical practice, the suicide attempt code in itself has often been considered sufficient to indicate distress when reported to the national hospital discharge register. A proportion of the reference subjects might, however, have had milder forms of psychiatric illness. Thus our reference group consisted of people who had attempted suicide and had none, subclinical, or milder forms of psychiatric morbidity at the index episode and within one year thereafter.

We studied eight psychiatric disorders: schizophrenia (code 295, international classification of diseases, eighth revision), bipolar and unipolar disorder (296.1-296.9), other depressive disorder (296.0, 300.4), anxiety disorder (300, except 300.4), adjustment disorder or post-traumatic stress disorder (307), alcohol abuse or dependence (303), drug abuse or dependence (304), and personality disorder (301). The hospital discharge register has national coverage for psychiatric disorders from 1973. Reporting to this register is mandatory for all healthcare providers, including the few existing private hospitals. Swedish registers are of good quality,^17^ and the validity of diagnoses for schizophrenia in the hospital discharge register was confirmed during a file based review by psychiatrists.^18^ For all disorders we used the principal diagnosis. As the cause of death register covers more than 99% of deaths in Swedish residents, including those occurring outside Sweden, the loss of information on death by suicide was minimal.^19^ We introduced the potential confounders of age, educational level, and immigrant status as covariates in the regression analyses. We measured education on a seven point ordinal scale from not having completed compulsory school (shorter than nine years) to postgraduate education, using the 1970 population and housing census and the education register for 1990, 2000, and 2004. Immigrant status (yes or no) was obtained from the migration register.

### Analyses

Patients were followed from discharge after attempted suicide to a definite or uncertain suicide (E950-9 and E980-9, international classification of diseases, eighth and ninth revisions, and X60-84 and Y10-34, ICD-10), death other than suicide, first emigration, or end of follow-up (31 December 2003). We included uncertain suicides because their exclusion might have led to an underestimation of suicide rates.^20^ Thus we followed-up patients for 21-31 years.

We used Kaplan-Meier survival curves to plot temporal patterns of suicide after a suicide attempt; we excluded adjustment disorder or post-traumatic stress disorder owing to absence of risk effect and substance misuse owing to low prevalence. For each diagnostic category and sex we determined separately absolute and relative mortality from suicide after a suicide attempt and used Cox regression models to compute hazard ratios (95% confidence intervals) taking time at risk into account. We adjusted the hazard ratios for age, highest level of education, and immigrant status. For each psychiatric disorder we calculated the population attributable fractions of suicide among people who had previously attempted suicide using the formula P×(HR−1/HR), where HR is the hazard ratio derived with Cox regression (adjusted for age, education, and immigrant status) and P the base rate of the disorder among all people who had completed suicide. We used SPSS for Windows (version 15) for the statistical analyses.

## Results

Death from suicide occurred primarily within the first five years after the index suicide attempt for all diagnostic groups, including those without a psychiatric diagnosis, but the risk prevailed over the total follow-up period (figure[Fig fig1]). A high proportion of all suicides in each diagnostic group occurred within the first year (figure[Fig fig1] and table[Table tbl1]). People who had attempted suicide and had a diagnosis of schizophrenia or bipolar and unipolar disorder had a highly increased risk, especially in the short term. For bipolar and unipolar disorder, 64% of suicides in men and 42% in women occurred within the first year; the corresponding proportions for schizophrenia were 56% and 54%. The total mortality from suicide in the reference group was comparatively low, although 45% of suicides in men and 40% in women took place within the first year. Schizophrenia and bipolar and unipolar disorder conferred the highest risks for suicide during the entire follow-up; hazard ratios adjusted for age, education, and immigrant status ranged from 2.5 for bipolar and unipolar disorder in women to 4.1 for schizophrenia in men (table[Table tbl1]). Patients with most other disorders had lower, but still significantly increased, risks for suicide. Those with adjustment disorder or post-traumatic stress disorder and alcohol misuse (men only), however, did not have significantly increased risks.

**Figure fig1:**
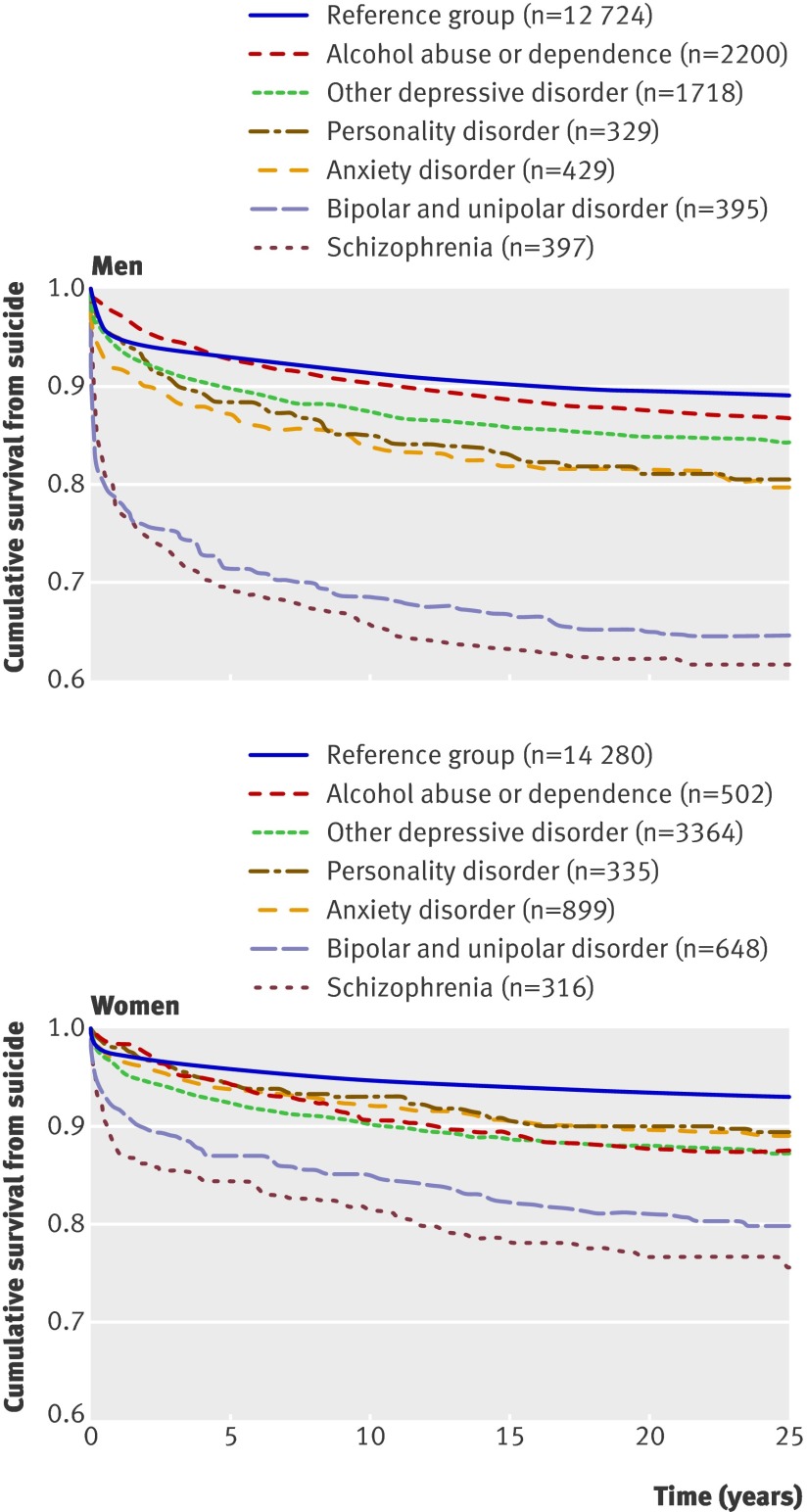
Survival graphs for suicide by psychiatric disorder in people admitted to hospital during 1973-82 for attempted suicide in Sweden and followed to 2003

**Table tbl1:** Absolute rates, hazard ratios, and population attributable fractions for death from suicide by psychiatric disorder in 39<thin>685 people who attempted suicide and were admitted to hospital during 1973-82 in Sweden and followed to 2003

Diagnosis	Mean (SD) age at suicide attempt (years)	Completed suicide within one year after suicide attempt			Completed suicide during entire follow-up		
		Suicide rate (%) (No)	Proportion of all suicides during follow-up (%)		Suicide rate (%) (No)	Adjusted hazard ratio* (95% CI)	Population attributable fraction
Bipolar and unipolar disorder:							
Male (n=395)	49.1 (16.4)	22.8 (90)	63.8		35.7 (141)	3.5 (3.0 to 4.2)	4.0
Female (n=648)	48.2 (15.4)	8.5 (55)	42.3		20.1 (130)	2.5 (2.1 to 3.0)	4.1
Other depressive disorder:							
Male (n=1718)	40.4 (15.3)	6.0 (103)	37.7		15.9 (273)	1.4 (1.2 to 1.6)	3.1
Female (n=3364)	40.1 (15.7)	4.0 (135)	31.4		12.8 (430)	1.7 (1.5 to 1.9)	9.3
Schizophrenia:							
Male (n=397)	34.1 (11.8)	21.7 (86)	56.2		38.5 (153)	4.1 (3.5 to 4.8)	4.6
Female (n=316)	38.7 (13.1)	13.0 (41)	53.9		24.1 (76)	3.5 (2.8 to 4.4)	2.8
Anxiety disorder:							
Male (n=429)	35.0 (13.2)	8.2 (35)	41.2		19.8 (85)	1.9 (1.5 to 2.3)	1.6
Female (n=899)	34.9 (14.1)	3.3 (30)	30.3		11.0 (99)	1.5 (1.3 to 1.9)	1.7
Adjustment disorder or post-traumatic stress disorder:							
Male (n=244)	36.1 (14.6)	1.6 (4)	17.4		9.4 (23)	0.9 (0.6–1.3)	−0.1
Female (n=520)	34.0 (14.4)	1.3 (7)	20.0		6.7 (35)	1.0 (0.7–1.4)	0.0
Alcohol abuse or dependence:							
Male (n=2200)	41.2 (12.2)	2.7 (60)	20.4		13.4 (294)	1.1 (1.0–1.3)	1.1
Female (n=502)	39.2 (12.1)	1.8 (9)	14.3		12.5 (63)	1.7 (1.3–2.1)	1.4
Drug abuse or dependence:							
Male (n=206)	32.2 (14.1)	2.4 (5)	13.5		18.0 (37)	1.6 (1.1–2.2)	0.6
Female (n=179)	34.8 (15.8)	2.8 (5)	16.7		16.8 (30)	2.3 (1.6–3.3)	0.9
Personality disorder:							
Male (n=329)	31.8 (11.1)	5.2 (17)	26.2		19.8 (65)	1.8 (1.4–2.3)	1.2
Female (n=335)	30.8 (12.3)	2.1 (7)	19.4		10.7 (36)	1.5 (1.1–2.1)	0.6
Reference group:							
Male (n=12 724)	37.9 (17.4)	5.1 (643)	45.1		11.2 (1426)	Reference	Reference
Female (n=14 280)	36.0 (17.6)	2.8 (402)	39.6		7.1 (1015)	Reference	Reference

Diagnoses were principal diagnoses assigned during an inpatient episode starting within one week after index suicide attempt.

*Hazard ratio for each coexistent psychiatric disorder compared with reference group (without any coexistent psychiatric disorder) category obtained with Cox regression modelling over total follow-up period. Hazard ratios were adjusted for age, educational level, and immigrant status.

The highest population attributable fractions for suicide among people who had previously attempted suicide were for other depression in women (9.3), followed by schizophrenia in men (4.6), and bipolar and unipolar disorder in women and men (4.1 and 4.0, respectively).

## Discussion

Type of coexistent psychiatric morbidity in people who have previously attempted suicide is related to risk of eventual death from suicide. To inform psychiatric and suicide prevention practices in the identification of people with a particularly high risk of suicide we investigated the longitudinal risk for completed suicide in patients who had attempted suicide as a function of eight coexistent psychiatric disorders. We found substantial differences in suicide risk across the diagnostic categories. The rate of suicide after a previous suicide attempt was particularly increased among men and women with schizophrenia or bipolar and unipolar disorder. Population attributable fractions, expressing the impact of coexistent psychiatric disorder on suicide in people who had previously attempted suicide, suggested a modest but significant impact for other depression in women, schizophrenia in men, and bipolar and unipolar disorder in both sexes. The larger population attributable fractions for these reflect their respective prevalence and the associated relative risk of suicide in the study cohort. Thus other depressive disorder had a comparatively high impact owing to its high prevalence, despite the risk of completed suicide not being as high as for schizophrenia or bipolar and unipolar disorder. In agreement with findings from the general population presented in a Danish study,^11^ our results show that affective disorders have a substantial impact on risk of suicide among people with previous suicidal behaviour. Importantly, as we only studied patients with previous suicidal behaviour and controlled for age, education, and immigrant status or ethnic minority group, hazard ratios and population attributable fractions in the present study were related to risks contributed by the mental disorder itself, or possibly some unmeasured confounders.

Death from suicide was heavily skewed towards the first years after the suicide attempt particularly in people with schizophrenia or bipolar and unipolar disorder, probably because of intense, symptom rich phases. Recent reviews suggest that suicide rates are about 10-fold higher for patients with schizophrenia and 20-fold higher for those with bipolar disorder than in the general population^21^ ^22^; the increased risks also seem to persist in comparisons between earlier and more recent cohorts.^22^ ^23^ ^24^ Risk factors for suicide in patients with schizophrenia^25^ or bipolar disorder^26^ have been summarised. The present results suggest that attempted suicide in those with schizophrenia or bipolar and unipolar disorder is particularly worrying and underlines the need for more focused care during at least the first two years after a suicide attempt.

We expected a relatively high impact of substance misuse or dependence on risk of suicide in people who had previously attempted suicide.^1^ The hazard ratio, however, exceeded 2.0 only in females with drug misuse or dependence who had attempted suicide. Yet we cannot rule out the possibility that forensic pathologists might be less likely to classify the cause of death as suicide in people with physical stigma of substance misuse; this would lead to misclassification and deflated risk estimates for suicide. Moreover, alcohol misuse could be comorbid with several disorders judged as principal, and thereby contribute to risk of completed suicide. Anxiety disorder conferred a comparably increased risk for suicide in both sexes. Importantly, a complicating depression may be instrumental in risk of suicide in people with anxiety disorder.^27^

By using an epidemiological framework and a total population sample we tried to minimise the selection bias and power problems in previous studies of smaller clinical samples. The national cohort we followed for at least 21 years is the largest with data on people who have attempted suicide and on psychiatric morbidity. Because the cause of death register has excellent coverage, loss to follow-up should be minimal. One limitation of our study was that we included only people with suicide attempts that led to an episode of inpatient care. Furthermore, we did not study the contribution of physical illness^4^ ^28^ and multiple psychiatric comorbidity. We did not analyse subcategories of the diagnostic groups because of the small numbers of suicides in some subgroups. Adjustment disorder or post-traumatic stress disorder was infrequently diagnosed in Swedish psychiatry during the inclusion period (1973-82) and the actual size of this group might be underestimated. Consequently our results for adjustment disorder or post-traumatic stress disorder should be interpreted with caution. Also, a narrow definition for bipolar disorder was used in Sweden during the years of inclusion; primarily for patients with more obvious manic symptoms and similar to the type I diagnosis for bipolar disorder by the *Diagnostic and Statistical Manual of Mental Disorders*, fourth edition. Thus these results might not be generalisable to a broader phenotype for bipolar disorder. Furthermore, among the disorders labelled as manic depressive in the international classification of diseases, eighth revision, the depressed type (296.2), which could be labelled recurrent severe depression, contributed strongly to the high risk of suicide in the bipolar and unipolar group. Also, we defined coexistent psychiatric morbidity as any disorder diagnosed within one week of the suicide attempt. People who attempted suicide may have been diagnosed as having one or more psychiatric disorders before or after this period, thereby resulting in misclassification of patients with coexistent psychiatric morbidity as reference subjects. Therefore we tested the effect of an alternative definition of reference subjects on estimates of suicide risk across diagnostic categories. The exclusion from the reference group of those receiving a psychiatric diagnosis beyond one year after the suicide attempt yielded similar hazard ratios and population attributable fractions across the diagnostic groups (data not shown). It is most likely that many subjects in the reference group had subclinical psychiatric morbidity. Our estimates are therefore probably an underestimation of the true risks conveyed by coexistent psychiatric morbidity in people who attempted suicide.

Specific treatment of patients who have attempted suicide is often discussed on the basis of previous suicide attempts^29^ and an estimate of suicidal intent. Similar to suggestions for young people who attempt suicide,^30^ ^31^ our results imply that interventions should take into account coexistent mental disorder and the time that has elapsed since the previous suicide attempt.

### Conclusion

In people who have attempted suicide the type of psychiatric morbidity and how recently the suicide was attempted should be considered during the clinical evaluation. Psychiatric case management should focus on more intensive aftercare during at least the first two years after a suicide attempt in patients with coexistent bipolar and unipolar disorder or schizophrenia. Further studies are needed to identify the characteristics of bipolar and unipolar disorder, schizophrenia, and other disorders, including comorbidity, that confer a higher risk for completed suicide.

**What is already known on this topic**

A previous suicide attempt is a well established risk factor for completed suicide
The impact of coexistent psychiatric morbidity on risk of suicide after suicide attempts is largely unknown

**What this study adds**

Schizophrenia and bipolar and unipolar disorder substantially influence overall risk and temporality for completed suicide after a suicide attempt in both sexes
Schizophrenia, bipolar and unipolar disorder, and other depression have the strongest population impact on risk of completed suicide in people who have previously attempted suicide

## Notes

We thank Eva Carlström for data management.

Ethical approval: This study was approved by the regional ethics committee at Karolinska Institutet.

All authors declare, as researchers, independence from the funders.

Provenance and peer review: Not commissioned; externally peer reviewed.

## Notes

**Cite this as: ***BMJ* 2008;337:a2205
